# Internal and external silica dust exposure threshold as an early screening index for silicosis: a cross-sectional study

**DOI:** 10.3389/fpubh.2025.1652017

**Published:** 2025-09-24

**Authors:** Shupeng Liu, Hailan He, Lei Chu, Qiong Song, Yuesong Zhai, Fang Yang, Heliang Liu

**Affiliations:** ^1^School of Public Health, North China University of Science and Technology, Tangshan, China; ^2^Hebei Key Laboratory of Organ Fibrosis, North China University of Science and Technology, Tangshan, China; ^3^Zibo Infectious Diseases Hospital, Zibo, China

**Keywords:** silicosis, cumulative dust exposure, blood silicon, urinary silicon, biological detection threshold

## Abstract

**Introduction:**

Common occupational hazards such as lead, chromium, and mercury have clear biological detection thresholds, but silicon dioxide does not. Therefore, this study aims to determine silica dust exposure thresholds internal and external exposure to identify early screening markers and help screening for susceptible individuals and prevent silicosis.

**Methods:**

Basic information, including physical examination data and questionnaires, was collected from the study participants. Blood and urine samples from iron mine workers were also collected as biological specimens. Silicon levels in these samples were measured using ICP-MS, and cumulative dust exposure was calculated based on an on-site hygiene investigation of the mine.

**Results:**

Overall, 1,964 participants were included in the study: 1,823 in the dust exposure group (without illness) and 141 in the silicosis group (with illness). Analysis revealed that the silicosis group had higher cumulative dust exposure, indicating an elevated external exposure index. Internal exposure indicators, such as elevated blood and urine silicon levels, were identified as risk factors for silicosis. Screening thresholds were determined using receiver operating characteristic curves and restricted cubic splines. The results showed that workers had an average dust exposure duration of 8.5 years. The threshold values were 8.02 μg/L for blood silicon with an area under the curve (AUC) of 0.557, 9.51 μg/L for urine silicon with AUC of 0.647, and 3728.50 mg·years for cumulative dust exposure with AUC of 0.658. When validated with external data from silica-exposed workers, blood silicon had the highest accuracy as an early screening indicator for silicosis.

**Conclusion:**

Blood silicon, urine silicon, and cumulative dust exposure were initially proposed as early screening indicators for silicosis. Validation with external worker data showed that blood silicon had relatively high threshol reliability as a screening marker.

## 1 Introduction

Silicosis is a systemic disease characterized by nodular lung fibrosis, mainly caused by prolonged inhalation of dust particles rich in free silicon dioxide (SiO_2_). It is a common form of pneumoconiosis. The pathogenesis of silicosis remains unclear, and no effective treatment exists (1–4). Therefore, early screening of susceptible populations and prompt removal from dusty environments are crucial for preventing and treating silicosis (5).

According to the Chinese occupational exposure limits for hazardous agents in the workplace - Part 1: Chemical hazardous agents (GBZ 2.1–2019), clear biological detection thresholds exist for common occupational hazards, such as blood lead, blood chromium, urine chromium, and urine mercury. However, no such threshold exists for silicosis. Previous research shows that silicon levels in the blood and urine of workers exposed to silica dust rise rapidly during the early stages of exposure, peak within 4–6 years, and then decline before stabilizing (6). These changes in silicon content reflects the direct manifestation of silicon dioxide in the body, it naturally precedes indirect reactions such as those of cytokines, highlighting the value of blood and urine silicon as early indicators of silicosis (6–8). Additionally, studies show that cumulative silica exposure (mg·a) is significantly and positively correlated with the cumulative incidence of pneumoconiosis (%), underscoring its significance in early diagnosis and screening of high-risk populations (9). However, the specific exposure threshold remains to be determined.

In this study, the silicon content was measured to represent internal exposure, and the cumulative dust exposure (CDE) was calculated to represent external exposure. Restricted cubic splines (RCS) and receiver operating characteristic (ROC) curves were used to determine the threshold for each screening indicator. So, this study aims to preliminarily identify early screening indicators and biological thresholds for silicosis, providing a theoretical basis for screening high-risk populations and preventing the disease among dust-exposed workers.

## 2 Materials and methods

### 2.1 Study participants

In total, 2,119 workers exposed to silica dust who underwent occupational health examinations between March 2023 and June 2024 were initially recruited. After excluding individuals with other lung, endocrine, or metabolic diseases, and those who have taken antioxidant drugs such as vitamin C 1 month before the physical examination, prior exposure to other toxic agents before employment at the iron mine, and incomplete responses, 1,964 participants met the inclusion criteria, yielding a response rate of 92.69%. Of these, 1,823 did not have silicosis (102 in the silica dust-free control group and 1,721 in the exposed non-silicosis group). The remaining 141 had silicosis (stage I, II, or III), resulting in a prevalence rate of 7.18%. All participants provided written informed consent. The study was approved by the Ethics Committee of North China University of Science and Technology (Approval Number: 2021041).

### 2.2 Data collection

Sociodemographic data, including sex, age, marital status, and educational level, were collected, along with information on behaviors and lifestyle (e.g., alcohol use and smoking), personal and family medical history, and detailed occupational history (e.g., job type, work location, position, employment date, working hours, and exposure to harmful occupational factors). Physical examination results (e.g., height, weight, lung function, blood tests, and urinalysis) were also recorded. In addition, enterprise-level information such as production processes, environmental monitoring, and general company conditions was collected.

### 2.3 Determination of silicon content

Silicon levels in blood and urine samples were measured using ICP-MS, following procedures detailed in our previous study (6). All operations were conducted under silicon-free conditions.

### 2.4 Determination of dust concentration

Total dust concentration, particle dispersion, and free silica content in settled workplace dust were measured using the filter membrane gravimetric method, in accordance with Chinese standard GBZ/T192.1–2007.

### 2.5 Sampling methods of air dust sample

Sampling methods of air dust sample adopts a combination of personal sampling and fixed-point sampling. In accordance with the requirements of Chinese “Sampling Specifications for the Detection of Hazardous Substances in the Air of Workplaces,” sampling should be conducted separately based on the type, nature, fluctuation situation and degree of hazard. Dust concentration determination: Set up dust detection points. Each detection point should be measured 1 to 3 times per shift for 2 consecutive days. The workers wore individual dust samplers and measured for two work shifts. Fixed-point sampling was carried out using the AKFC-92A dust sampler (Shanghai Yichang Industrial Co., Ltd. China). The sampling filter membrane was a mixed cellulose ester filter membrane with a diameter of 37 mm and a pore size of 0.8 μm, with a flow rate of 5 L/min. The flow rate of the individual sampler is 2 L/min, collecting air samples from the entire shift of the workers (8 h). As shown in Supplementary Table 1.

### 2.6 Calculation of cumulative dust exposure

Each worker’s dust exposure years were calculated based on the start and end dates (employment time) of each job type and associated exposure to harmful factors recorded in their occupational history. CDE was then estimated by job type using the following formula:


$$\mathrm{CDE}=\sum_{j}\;\sum_{\mathrm{ds}}^{\mathrm{de}}\;S_{\mathrm{jt}}$$

The unit of CDE is mg/m^3^·years. S_jt_ is the average annual exposure measurement for job type *t* in workshop *j* during year *TTH*, ds is the year the worker began job type *t* in workshop *j*, and de is the year the worker ended certain job type *t* in workshop *j* (For silicosis patients, *de* is the year diagnosed with silicosis). CDE reflects both the duration and intensity of dust exposure (10).

### 2.7 Statistical analysis

Statistical analyses were conducted using SPSS 23.0 and SAS 9.4. Normally distributed quantitative data are expressed as mean ± standard deviation, while categorical variables are described as composition ratios. Pearson correlation, Spearman correlation, and chi-square test were used for correlation analyses of normally distributed, non-normally distributed, and categorical data, respectively. ROC curves and RCS were used to identify screening thresholds. All hypothesis tests were two-sided, with a significance level of *α* = 0.05.

## 3 Results

### 3.1 Basic characteristics of the study participants

All participants were male and were classified into non-silicosis and silicosis groups based on disease status. Potential risk factors for silicosis in dust-exposed workers, including age, smoking, alcohol consumption, body mass index (BMI), and forced expiratory volume in 1 s (FEV1%), were described and compared. Significant differences were observed between the two groups regarding age, drinking, BMI, FEV1%, and years of dust exposure (*p* < 0.05), while no difference was found in smoking status (Table 1).

**Table 1:** Characteristics of the study participants.

Variable		Total (*n*, %)	Non-silicosis group (*n*, %)	Silicosis group (*n*, %)	*p-*value
Age (years)	<30	78 (3.97)	77 (4.21)	1 (0.70)	<0.05
	30–40	402 (20.47)	389 (21.32)	13 (9.21)	
	40–50	719 (36.61)	672 (36.88)	47 (33.32)	
	>50	765 (38.95)	685 (37.19)	80 (56.67)	
Smoking	Non-smoker	726 (36.97)	672 (36.88)	54 (38.32)	0.26
	Smoker	1,084 (55.19)	1,003 (55.01)	81 (57.41)	
	Quit smoking	154 (7.84)	148 (8.11)	6 (4.27)	
Drinking	No drinking	596 (30.35)	530 (29.11)	66 (46.76)	<0.05
	Drinking	1,292 (65.78)	1,221 (67.02)	71 (50.42)	
	Stop drinking	76 (3.87)	72 (3.87)	4 (2.82)	
BMI (kg/m^2^)	Underweight	3 (0.15)	3 (0.21)	0	<0.05
	Normal	738 (37.58)	699 (38.31)	39 (27.68)	
	Overweight	883 (44.96)	821 (45.88)	62 (44.01)	
	Obese	340 (17.31)	300 (16.50)	40 (28.41)	
FEV1%	80%	1788 (91.04)	1,680 (92.21)	108 (76.62)	<0.05
	70–80%	140 (7.13)	109 (6.00)	31 (22.02)	
	70%	36 (1.83)	34 (1.89)	2 (1.36)	
Dust exposure years (years)	10	783 (39.87)	757 (41.51)	26 (18.44)	<0.05
	10–20	674 (34.31)	637 (34.91)	37 (26.21)	
	20–30	333 (16.96)	305 (16.68)	28 (19.87)	
	30	174 (8.86)	124 (6.80)	50 (35.46)	

BMI, body mass index; FEV1%, forced expiratory volume in 1 s.

### 3.2 Dust exposure overview

The primary job categories in the mine include mining, crushing, grinding and selection, as well as auxiliary operations. Based on dust exposure data from 2021 to 2024, an analysis of variance revealed significant differences among job types, except for auxiliary roles (*p* < 0.01; Table 2).

**Table 2:** Silica dust exposure from 2021 to 2024 (mg/m^3^·day).

Job type	2021	2022	2023	2024	*p-*value
Mining	1.88 ± 0.15	0.71 ± 0.13	0.44 ± 0.04	0.7 ± 0.25	0.004
Crushing	4.6 ± 0.95	0.81 ± 0.16	0.86 ± 0.2	0.82 ± 0.25	<0.001
Grinding and selection	1.22 ± 0.3	0.91 ± 0.09	0.85 ± 0.11	0.35 ± 0.02	<0.001
Auxiliary	0.28 ± 0.04	0.2 ± 0.77	0.13 ± 0.03	0.25 ± 0.06	0.174

### 3.3 Comparison of cumulative dust exposure and silicon levels

CDE, blood silicon, and urine silicon levels were compared among the dust-free control, dust exposure, and silicosis groups. Analysis of variance revealed significant differences across all groups (*p* < 0.01; Table 3).

**Table 3:** Comparison of internal and external exposure levels under different silica dust exposure conditions.

Item	Control group (*n* = 102)	Dust exposure group (*n* = 1721)	Silicosis group (*n* = 141)	*p*
Cumulative dust exposure (mg·years)	0	2487.23 ± 235.75^a^	2926.46 ± 240.03^a^	<0.01
Blood silicon value (μg/mL)	5.28 ± 1.68	9.80 ± 1.67^a^	9.55 ± 3.55^a^	<0.01
Urine silicon value (μg/mL)	6.24 ± 1.70	12.29 ± 1.86^a^	13.39 ± 4.80^ab^	<0.01

^a^ Compared with the control group, *p* < 0.05; ^b^ Compared with the silica dust-exposed group, *p* < 0.05; ^c^ Compared with the silicosis group, *p* < 0.05.

### 3.4 Correlation between cumulative dust exposure and silicon levels

Correlation analysis was conducted between internal exposure indicators (blood and urine silicon levels) and the external exposure indicator (CDE). Both blood and urine silicon levels showed a significant positive correlation with CDE (*p* < 0.01). The correlation with urine silicon was strong, while that with blood silicon was moderate (Table 4).

**Table 4:** Cumulative dust exposure and urinary and blood silicon levels.

Cumulative dust exposure	*n*	*r*	*p*
Blood silicon value (μg/mL)	1964	0.42	<0.01
Urine silicon value (μg/mL)	1964	0.66	<0.01

### 3.5 Analysis results of early screening indicators for silicosis

**Figure 1:**
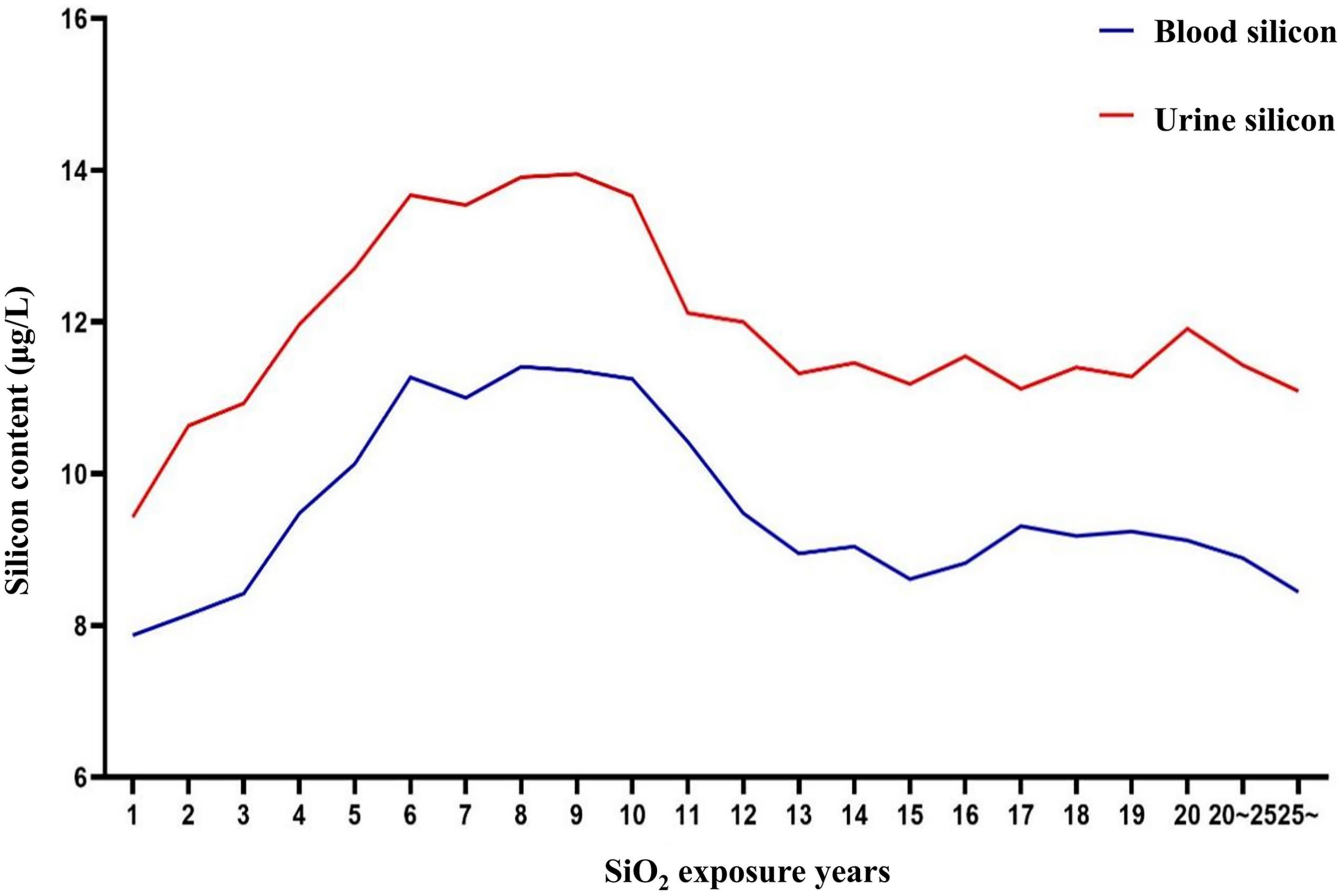
Relationship between dust exposure years and blood/urine silicon levels.

Blood and urine silicon levels (internal exposure), CDE (external exposure), and years of dust-exposed work were selected as early screening markers for silicosis. Thresholds for each screening index were identified using RCS and ROC curves. According to the relationship curves, blood and urine silicon levels rose rapidly within the first 1–6 years of exposure, stabilized between years 6 and 10, and then declined. Urine silicon levels peaked around year 9, while blood silicon levels peaked around year 10. These findings suggest that the highest risk period for developing silicosis occurs between 6 and 10 years after initial silica-dust exposure (Figure 1).

### 3.6 Silicon levels as an indicator of silicosis

RCS and the ROC curves were jointly used to determine thresholds for blood and urine silicon as screening indicators for silicosis. Since RCS did not identify clear thresholds, only the points corresponding to the maximum kappa values from the ROC curves were selected.

**Figure 2:**
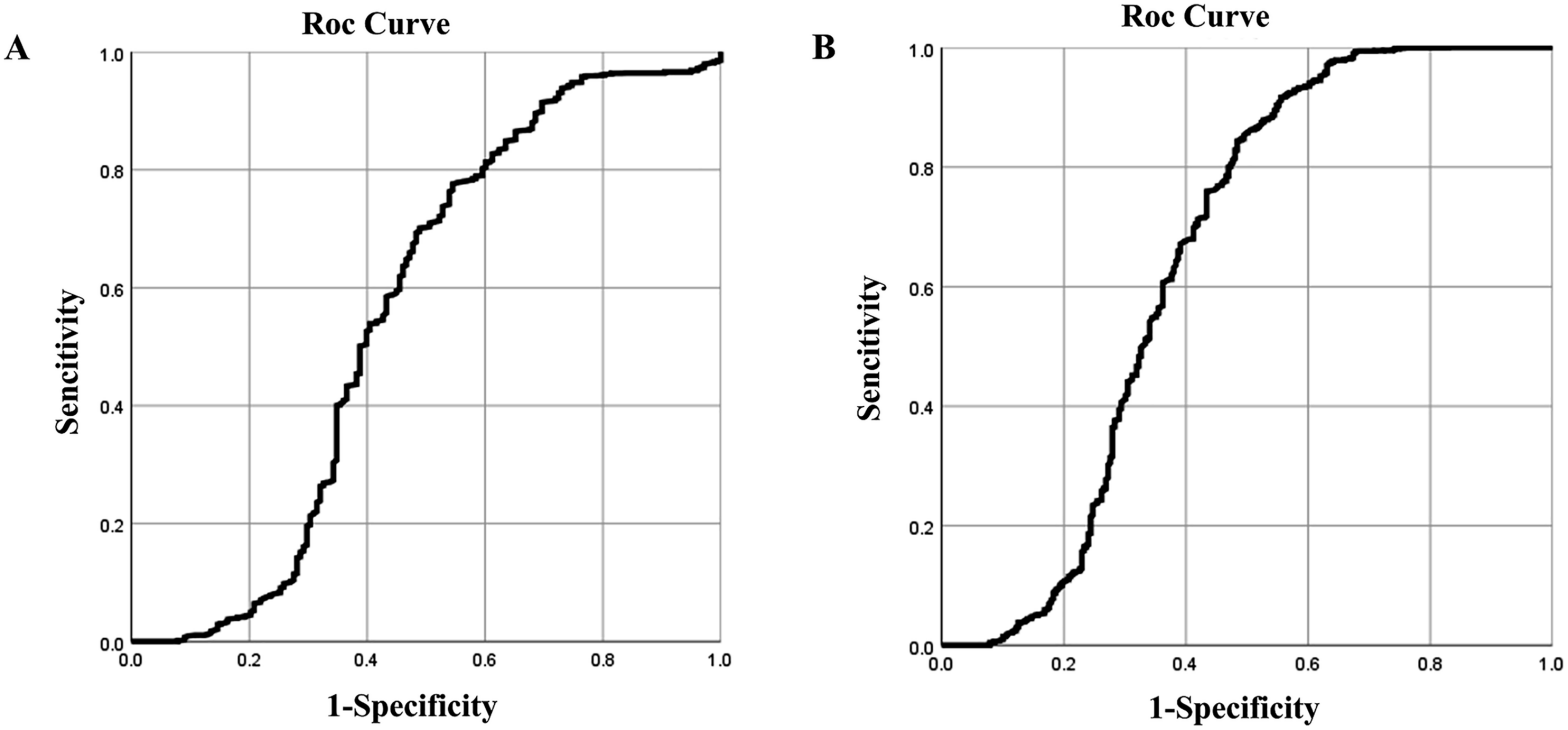
ROC curves of blood and urine silicon levels for silicosis screening. ROC curves for **(A)** blood; **(B)** urine silicon.

For blood silicon, the ROC analysis showed statistical significance (*p* < 0.05), with an area under the curve (AUC) of 0.557. Analysis showed that the optimal blood silicon threshold was 8.015 μg/L, corresponding to the highest kappa value, with sensitivity and specificity of 0.776 and 0.455, respectively (Table 5; Figure 2A).

**Table 5:** ROC curve results of blood silicon levels and silicosis.

AUC	SE	*p-*value	95% CI	
			Lower limit	Upper limit
0.56	0.03	0.01	0.5	0.62

AUC, area under the curve; ROC, receiver operating characteristics; CI, confidence interval; SE, Sensitivity.

For urinary silicon, ROC analysis revealed statistical significance (*p* < 0.05), with an AUC of 0.647. The optimal urinary silicon threshold was 9.51 μg/L, yielding the highest kappa value, with sensitivity and specificity of 0.917 and 0.444, respectively (Table 6; Figure 2B).

**Table 6:** ROC curve results of urine silicon levels and silicosis.

AUC	SE	*p-*value	95% CI	
			Lower limit	Upper limit
0.65	0.02	<0.01	0.60	0.69

AUC, area under the curve; ROC, receiver operating characteristics; CI, confidence interval; SE, Sensitivity.

The AUC of both blood silicon and urine silicon is relatively low, especially blood silicon with 0.56 AUC is marginal at best. Although it within the scope of statistics, external validation is still required to obtain the corresponding threshold.

### 3.7 External cumulative dust exposure as an indicator of silicosis

The ROC curve and RCS were used to determine the threshold for CDE as a screening indicator for silicosis, yielding values of 3207.235 mg·years and 4,250 mg·years, respectively. The average threshold was 3728.5 mg·years. Accordingly, the CDE among workers in this iron mine should not exceed 3728.5 mg·years.

### 3.8 ROC curve of cumulative dust exposure and silicosis

**Figure 3:**
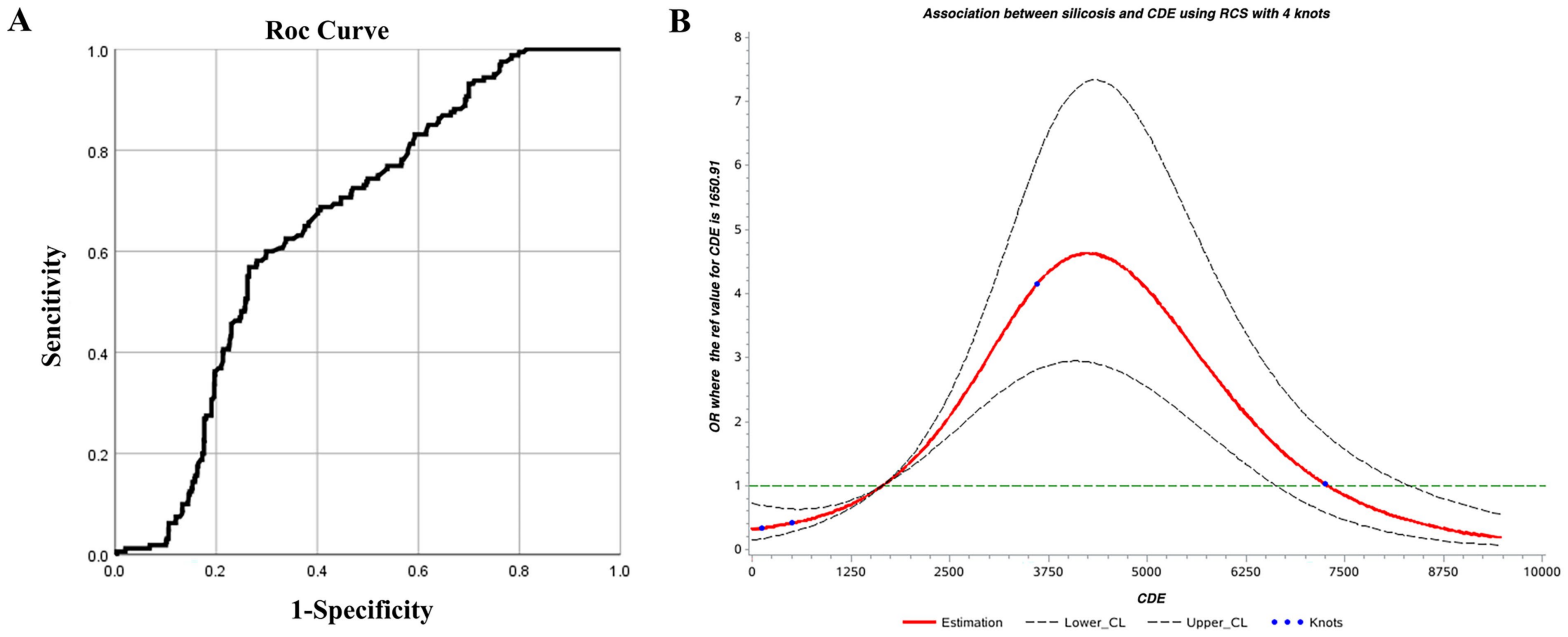
**(A)** ROC and **(B)** restricted cubic spline curves for cumulative dust exposure and silicosis.

The ROC curve was used to determine the threshold of CDE as a screening indicator for silicosis. The results were statistically significant (*p* < 0.05) with an AUC of 0.658, indicating acceptable screening performance. The optimal CDE threshold, corresponding to the kappa value, was 3207.235 mg·years, with sensitivity and specificity of 0.569 and 0.735, respectively (Table 7; Figure 3).

**Table 7:** ROC curve results of cumulative dust exposure and silicosis.

AUC	SE	*p*-value	95% CI	
			Lower limit	Upper limit
0.658	0.019	<0.001	0.622	0.694

AUC, area under the curve; ROC, receiver operating characteristics; CI, confidence interval; SE, Sensitivity.

### 3.9 Restrictive cubic splines of cumulative dust exposure and silicosis

Using an RCS model with four knots, the Akaike Information Criterion (AIC) of the model was 1090.70. CDE showed a nonlinear dose–response relationship with silicosis. The overall association was significant (*χ^2^* = 102.54, *p* < 0.01), as was the test for nonlinearity (*χ^2^* = 71.62, *p* < 0.01). Based on the odds ratio (OR) confidence interval, CDE between 1750 and 7,250 mg·years was associated with an increased risk of silicosis (OR >1). The risk peaked at approximately 4,250 mg·years, indicating a rapid increase in risk from 1750 to 4,250 mg·years, followed by a gradual decline between 4,250 and 7,250 mg·years (Figure 3). This phenomenon is quite interesting and seems contrary to common sense, it should be that the OR value increases with the increase of the CDE. However, as shown in this chart, the OR value does indeed decline after exceeding the peak of 4,250 mg·years. It is speculated that this is related to the susceptibility to silicosis. Before the peak, the risk of developing the disease is relatively high, while after the peak, since those who should have developed the disease have already done so, the remaining population is less susceptible to silicosis. Therefore, the OR value keeps decreasing. This phenomenon also deserves further study in the future.

Using CDE as a screening indicator for silicosis, thresholds identified by the ROC curve and RCS were 3207.235 and 4,250 mg·years, respectively. The average value of 3728.5 mg·years was adopted as 3728.5 mg·years as the screening threshold.

### 3.10 Dust exposure duration as a screening indicator for silicosis

Years of dust exposure are a simple, practical, and effective indicator for early screening of silicosis. Therefore, they were evaluated as a screening threshold using both the ROC curve and RCS, which yielded thresholds of 8.915 and 8 years, respectively. The average, 8.5 years, was adopted as the screening threshold. Accordingly, dust exposure among workers in this iron mine should not exceed 8.5 years.

### 3.11 ROC curve of dust exposure years and silicosis

**Figure 4:**
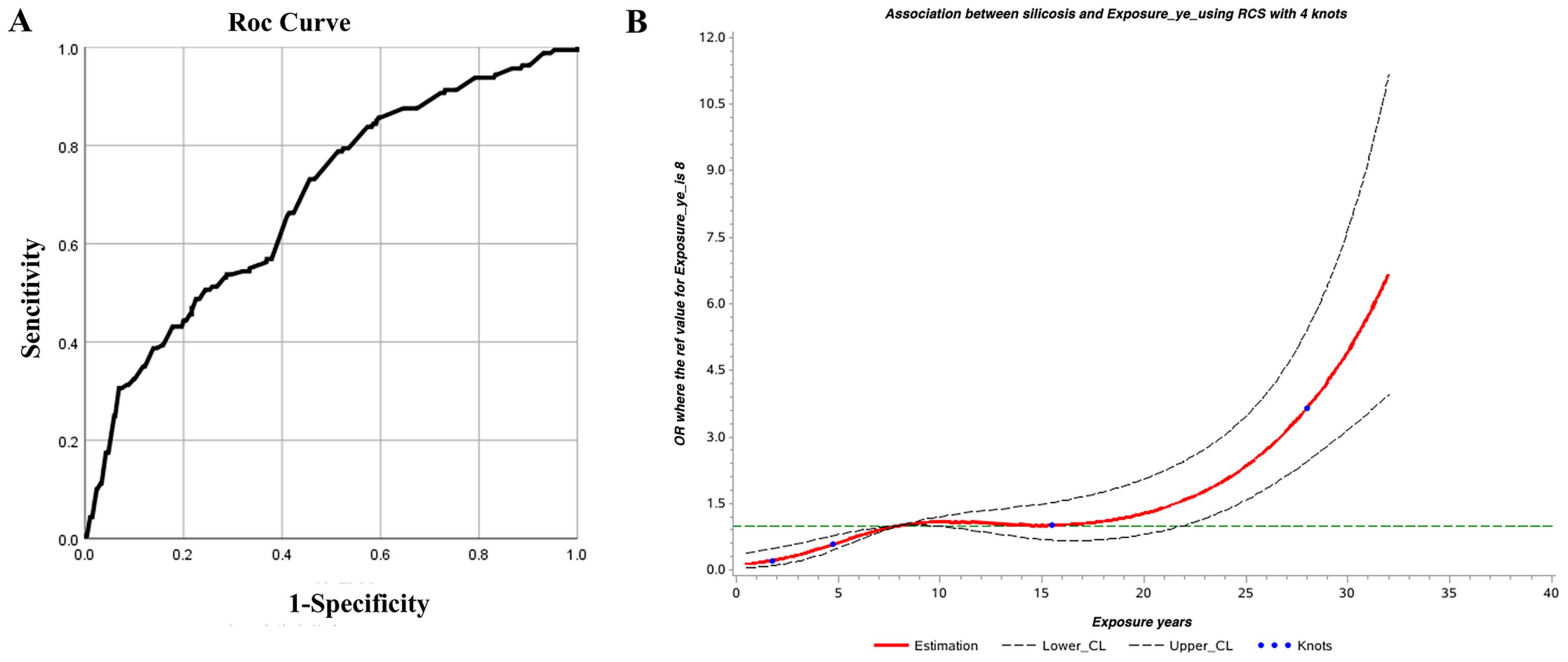
**(A)** ROC and **(B)** restricted cubic spline curves for dust exposure years and silicosis.

The ROC curve was used to identify the threshold of dust exposure years for silicosis screening. Results showed statistical significance (*p* < 0.05) with an AUC of 0.689, indicating acceptable screening performance. The optimal dust exposure threshold was 8.915 years, corresponding to the kappa’s highest value, with sensitivity and specificity of 0.73 and 0.54, respectively (Table 8; Figure 4).

**Table 8:** ROC curve results of dust exposure years and silicosis.

AUC	SE	*p*	95% CI	
			Lower limit	Upper limit
0.69	0.02	<0.01	0.65	0.73

AUC, area under the curve; ROC, receiver operating characteristics; CI, confidence interval; SE, Sensitivity.

### 3.12 Restricted cubic spline of dust exposure years and silicosis

Using the RCS model with four nodes, the AIC of the model was 1094.47. Dust exposure years showed a nonlinear dose–response relationship with silicosis. The overall association was significant (*χ^2^* = 82.50, *p* < 0.01), as was the test for nonlinearity (*χ^2^* = 10.19, *p* < 0.01). When the dust exposure period exceeds 8 years, the OR is greater than 1, indicating increased risk of silicosis. The OR remained stable between 8 and 15 years, then rose sharply beyond 15 years, suggesting a higher risk of silicosis (Figure 4).

### 3.13 Validation of external data

In total, 330 individuals were included in the validation set: 79 from a coal mine control group, 112 silica-exposed tunneling workers, and 139 cases of silicosis. ROC curve analysis of blood and urine silicon levels was conducted to assess the accuracy of previously identified screening indicators. Results showed that the blood silicon threshold slightly differed from that of iron mine workers but remained a relatively reliable screening marker for silicosis. In contrast, urine silicon levels showed greater variability, and their threshold requires further validation (Table 9).

**Table 9:** Blood and urine silicon concentration in the validation set/*M* (*P_25_*, *P_75_*).

Silica content /(μg/mL)	Groups			*H*	*p*
	Control group (*n* = 79)	Dust exposure group (*n* = 112)	Silicosis group (*n* = 139)		
Blood silicon	7.04 (6.4, 7.90)	11.69 (10.33, 13.44)	10.98 (10.24, 11.99)	70.75	<0.05
Urine silicon	8.03 (6.83, 9.03)	12.50 (9.88, 14.57)	10.16 (9.29, 11.69)	41.71	<0.05

**Figure 5:**
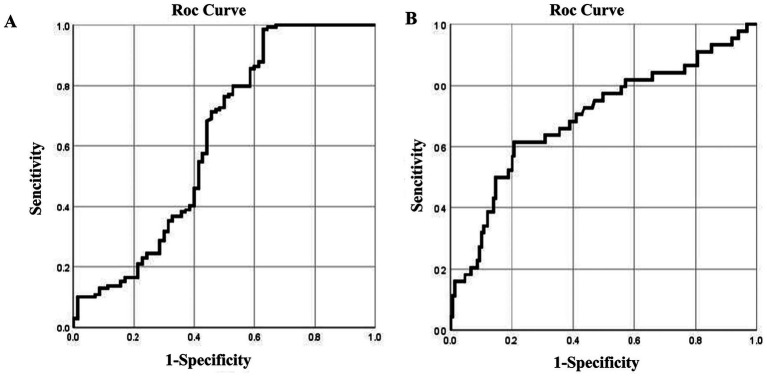
ROC curves of blood and urine silicon levels in the validation set for silicosis. ROC curves for **(A)** blood silicon and **(B)** urine silicon.

Validation of the blood silicon threshold using the ROC curve showed statistical significance (*p* < 0.05), with an AUC of 0.63. The optimal threshold, based on the highest kappa value, was 8.81 μg/L, with sensitivity and specificity of 0.99 and 0.37, respectively. This closely aligns with the previously identified threshold of 8.02 μg/L for iron ore dust-exposed workers, supporting the reliability of blood silicon as a screening marker for silicosis (Figure 5A).

Validation of the urine silicon threshold using the ROC curve showed significance (*p* < 0.05), with an AUC of 0.70. The optimal threshold, based on the highest kappa value, was 11.05 μg/L, with a sensitivity of 0.63 and specificity of 0.97. This value is notably higher than the 9.51 μg/mL threshold identified in iron ore dust-exposed workers, indicating that further validation is needed for urine silicon as a screening marker (Figure 5B).

## 4 Discussion

Silicosis, the most common type of pneumoconiosis, is the most prevalent occupational disease in China and among the most serious worldwide (11–13). The World Health Organization and International Labor Organization aim to eliminate pneumoconiosis by 2030 (14). Achieving this goal requires effective prevention of silica dust exposure and the identification of early biological screening thresholds. However, the Occupational Exposure Limits for Hazardous Factors in the Workplace: Part 1 of the Occupational Health Standards of the People’s Republic of China (GBZ 2.1–2019) provides no guidance on biological monitoring indicators or occupational exposure biological limits. Therefore, establishing biological monitoring indicators and occupational exposure limits for silica dust is urgently needed to guide occupational health standards.

Silicosis is progressive and irreversible, often leading to severe morbidity and mortality even after exposure ends. Clinical studies show that early diagnosis significantly improves survival in patients with silicosis, while advanced stages are associated with sharply increased mortality. The findings underscore the urgent need for systematic early screening, such as high-resolution computed tomography, and stricter occupational health regulations to reduce silica exposure (15, 16).

Several potential biomarkers for silicosis have been proposed, but none have been validated for clinical use. For example, IL-8 shows promise in detecting silicosis and predicting mortality (17), while serum CC16 and L-selectin levels may serve as viable alternatives. Neopterin levels in urine and serum have been linked to dust exposure, and lactate dehydrogenase may indicate silica-induced toxicity in agate workers. CXCL16 has emerged as a potential biomarker for distinguishing between silica exposure and silicosis (18–21). Blood inflammatory markers also correlate with silicosis and impaired lung function (22). Additionally, certain VOCs in exhaled breath appear suitable for identifying silica exposure (23, 24). Based on biological rationality, these biomarkers have good statistical significance in some studies. However, most initial biomarker studies suffer from limited and non-representative cohorts. While statistically significant results can be found in small groups, these findings may not be generalizable to the entire population at risk. In the meantime, due to limitations such as small sample size, specificity and sensitivity, and the high cost and difficulty in promotion of instruments and equipment, they have not yet been widely promoted in clinical practice and practical applications. Thus, further research with representative samples is needed to confirm their clinical utility.

We focus on SiO_2_, the primary hazard in silicosis. Traditionally, SiO_2_ is considered biologically inert. Once it enters the lungs, it remains deposited in the lesion or phagocytosed by alveolar macrophages without undergoing metabolism. However, studies show that trace amounts of silicon can enter the bloodstream and tissues, undergo metabolism, and exert toxic effects (6). Thus, silicon may indicate systemic SiO_2_ accumulation. Our previous research found that both blood and urine silicon levels reflect internal silicon burden. After exposure to silica dust, the silicon levels in the body change at an early stage, often before other recognized early biomarkers of silicosis (7, 8). Epidemiological studies suggest that silicosis can develop or progress even after exposure ends, indicating a threshold lung burden above which the disease progresses without further exposure (25). However, this threshold remains undefined.

Our previous measurements of silicon levels in workers exposed to silica dust showed a rapid increase during the first 1–5 years, followed by a decline in serum and urine silicon with prolonged exposure (6, 8). Similarly, animal studies found significantly elevated serum silicon levels in rats exposed to silica dust (7). After SiO_2_ enters the body, a compensatory clearance mechanism is triggered, increasing silicon metabolism and leading to an early metabolic peak. As exposure continues over time, the capacity of the body to metabolize SiO_2_ becomes saturated, entering a decompensated phase. Therefore, silicon levels gradually decline in the later stages of exposure, although the precise metabolic mechanism remains unclear.

Recent studies have identified a link between blood and urine silicon levels and the development of pulmonary fibrosis in both animals and humans. These findings suggest that blood and urine silicon may reflect early-stage pulmonary fibrosis and show changes earlier than traditional inflammatory markers such as TGF-β1, TNF-*α*, and CC16, highlighting their potential for early screening (26, 27). In addition, Fan et al. examined the dose–response relationship of pneumoconiosis using cumulative free silica exposure as an indicator across various dust types with different silica content (9). The results showed a significant positive correlation between cumulative silica exposure (mg·years) and the cumulative incidence of pneumoconiosis (%), underscoring the role of free silica content in pneumoconiosis pathogenesis. However, despite decades of research, few studies have examined the link between cumulative silica exposure and silicosis. Most have focused on immediate exposure limits, such as permissible exposure limits, which represent the average RCS exposure over an 8-h shift and vary from 0.05 (USA) to 0.35 mg/m^3^ (China) (28). Moreover, the average intensity of RCS exposure remains debated (2) and annual cumulative exposure thresholds were not addressed. Therefore, the existing CDE research mainly focuses on the dose–response relationship with silicosis but rarely involves the CDE threshold.

This study used CDE as the external exposure indicator and blood and urine silicon levels as internal exposure indicators. Thresholds were identified using the RCS and ROC curves. Both regression models for CDE were statistically significant, establishing 3728.5 mg·years as the preliminary screening threshold. Although RCS results for blood and urine silicon lacked statistical significance, external validation (29) confirmed blood silicon as a reliable indicator for early screening of silicosis. Theoretically, it is speculated that blood silicon should be more stable and reliable than urine silicon, urinary silicon is more susceptible to the influence of metabolism in the body, but the detection of urine silicon is simpler. In view of this study AUCs for blood and urine silicon are low, therefore these thresholds are preliminary and require prospective validation. It should also be emphasized that the determination of silicon requires strict “silicon-free” conditions. Additionally, better instruments and equipment are needed to eliminate determination bias.

This cross-sectional study analyzes silicosis among workers undergoing physical examinations from 2023 to 2024. However, due to the small sample size, this study did not conduct further analysis on factors such as age, educational level, smoking and drinking, and type of work. In the meantime, it lacks longitudinal data and is limited to a single iron mine without a large, multicenter sample and difficult to generalize to other scenarios and situations. So, this study merely made a preliminary attempt, providing a model for similar research in the future and playing a leading and exemplary role. When the sample size is expanded and different scenarios are applied in the future, more convincing results may be obtained.

In conclusion, blood and urine silicon levels can serve as early screening indicators for silicosis. CDE can be estimated using job type, exposure type, and duration of dust exposure. Years of dust exposure also offer predictive value. Once workers reach a high-risk exposure period, they should be reassigned from silica-exposed roles. Notably, these thresholds are intended solely for screening for susceptible individuals and not for diagnosing or assessing silicosis prognosis.

## Data Availability

The data analyzed in this study is subject to the following licenses/restrictions: the health check data of the enterprise staff cannot be made public. Requests to access these datasets should be directed to liuheliang@ncst.edu.cn.
