# Supplementary material for: Internal and external silica dust exposure threshold as an early screening index for silicosis: a cross-sectional study
Source: Front Public Health. 2025 Sep 24;13:1652017. doi: 10.3389/fpubh.2025.1652017 (PMC12504339; doi:10.3389/fpubh.2025.1652017)
Supplement: Supplementary file 1 [file Table_1.DOCX]

**Supplementary Table 1**. The setting of personal sampling numbers and detection points for silica dust

| Job type | Personal samples number | Fixed-point sampling location |
| --- | --- | --- |
| Mining | 4 | Drilling machine  Excavators, mining sites  Gun hammer cab |
| Crushing | 4 | Discharge port, duty room  Crusher, 1# belt head  Middle part of 1# belt and 2# belt head  2# belt end, Duty room  Medium and fine crusher, duty room  10# Belt, 11# Belt  belt12#, screening room |
| Grinding and selection | 7 | 104#ball grinder  The third floor magnetic separator  The fourth floor magnetic separator  The first floor main plant sand pump  vacuum pump  Sand screening machine, 2# pump room |
| Auxiliary | 2 | Equipment maintenance room  Electrician's duty room |

**Supplementary Table 2**. Determination results of free silica in the working environment of iron ore workers from 2021 to 2024

| Year | Job type | Free SiO_2_ content (%) |
| --- | --- | --- |
| 2021 | Mining | 23.46 |
|  | Crushing | 22.85 |
|  | Grinding and selection | 31.40 |
|  | Auxiliary | - |
| 2022 | Mining | 17.9 |
|  | Crushing | 12.74 |
|  | Grinding and selection | 10.22 |
|  | Auxiliary | - |
| 2023 | Mining | 20.7 |
|  | Crushing | 20.88 |
|  | Grinding and selection | 17.56 |
|  | Auxiliary | - |
| 2024 | Mining | 24.93 |
|  | Crushing | 25.34 |
|  | Grinding and selection | 21.06 |
|  | Auxiliary | - |

Note: In accordance with the "National Health Occupational Standards of the People's Republic of China" (GBZ2.1-2007) and the "National Health Occupational Standards of the People's Republic of China" (GBZ2.1-2019). –: Undetected.
